# Trophic effects of adipose-tissue-derived and bone-marrow-derived mesenchymal stem cells enhance cartilage generation by chondrocytes in co-culture

**DOI:** 10.1371/journal.pone.0190744

**Published:** 2018-02-28

**Authors:** M. M. Pleumeekers, L. Nimeskern, J. L. M. Koevoet, M. Karperien, K. S. Stok, G. J. V. M. van Osch

**Affiliations:** 1 Department of Otorhinolaryngology, Head and Neck surgery, Erasmus MC, University Medical Center, Rotterdam, the Netherlands; 2 Institute for Biomechanics, ETH, Zürich, Switzerland; 3 Department of Orthopaedics, Erasmus MC, University Medical Center, Rotterdam, the Netherlands; 4 Department of Tissue Regeneration, MIRA-institute for Biomedical Technology and Technical Medicine, University of Twente, Enschede, the Netherlands; Paracelsus Medical University, GERMANY

## Abstract

**Aims:**

Combining mesenchymal stem cells (MSCs) and chondrocytes has great potential for cell-based cartilage repair. However, there is much debate regarding the mechanisms behind this concept. We aimed to clarify the mechanisms that lead to chondrogenesis (chondrocyte driven MSC-differentiation versus MSC driven chondroinduction) and whether their effect was dependent on MSC-origin. Therefore, chondrogenesis of human adipose-tissue-derived MSCs (*h*AMSCs) and bone-marrow-derived MSCs (*h*BMSCs) combined with bovine articular chondrocytes (*b*ACs) was compared.

**Methods:**

*h*AMSCs or *h*BMSCs were combined with *b*ACs in alginate and cultured *in vitro* or implanted subcutaneously in mice. Cartilage formation was evaluated with biochemical, histological and biomechanical analyses. To further investigate the interactions between *b*ACs and *h*MSCs, (1) co-culture, (2) pellet, (3) Transwell® and (4) conditioned media studies were conducted.

**Results:**

The presence of *h*MSCs–either *h*AMSCs or *h*BMSCs—increased chondrogenesis in culture; deposition of GAG was most evidently enhanced in *h*BMSC/*b*ACs. This effect was similar when *h*MSCs and *b*AC were combined in pellet culture, in alginate culture or when conditioned media of *h*MSCs were used on *b*AC. Species-specific gene-expression analyses demonstrated that *aggrecan* was expressed by *b*ACs only, indicating a predominantly trophic role for *h*MSCs. *Collagen-10*-gene expression of *b*ACs was not affected by *h*BMSCs, but slightly enhanced by *h*AMSCs. After *in-vivo* implantation, *h*AMSC/*b*ACs and *h*BMSC/*b*ACs had similar cartilage matrix production, both appeared stable and did not calcify.

**Conclusions:**

This study demonstrates that replacing 80% of *b*ACs by either *h*AMSCs or *h*BMSCs does not influence cartilage matrix production or stability. The remaining chondrocytes produce more matrix due to trophic factors produced by *h*MSCs.

## Introduction

Cartilage has a very limited capacity for self-regeneration. Untreated lesions—caused by trauma, tumors, congenital malformation or age related degeneration—persist indefinitely and ultimately require surgical intervention. However, current treatments are unsuccessful for long-term repair; resulting in a need for novel repair strategies. Cell-based cartilage repair holds promise for restoring missing or destroyed cartilage and has the potential to overcome limitations of current treatments, while re-establishing the unique biological and functional properties of the tissue.

One of the major challenges herein is defining an appropriate cell source. Current cell-based surgical treatments for cartilage lesions are predominantly based on the use of either (1) chondrocytes or (2) mesenchymal stem cells (MSCs). These cell-based procedures are however associated with specific disadvantages. Chondrocytes from several anatomical locations (e.g. joint, rib, nose, ear, meniscus) have been investigated for their application in cartilage regeneration. [1–21] However, to generate a construct of reasonable size, large numbers of chondrocytes are required, necessitating the use of culture-expansion. In monolayer culture-expansion, chondrocytes dedifferentiate; they change phenotypically to a fibroblast-like morphology and lose their chondrogenic gene-expression capacity. Chondrocyte-dedifferentiation usually results in fibrous and mechanically inferior cartilage, making them less suitable for cell-based cartilage repair. [22] In contrast, multipotent cells, like MSCs, achieved considerable attention as alternative cells, as they can undergo multiple population doublings without losing their chondrogenic potential and have the capacity to differentiate into cartilage tissue under appropriate culture conditions. [23–27] Furthermore, MSCs are easily available from several tissues, including bone marrow and adipose tissue, which makes culture-expansion unnecessary. However, the single use of MSCs for cell-based cartilage repair is currently debated, since the cartilage tissue formed is unstable and predisposed to mineralization and ossification *in vivo*. [28–32]

Currently, combining both cell sources holds great promise for cell-based cartilage repair as it reduces the required number of chondrocytes and diminishes many disadvantages of both individual cell types. Moreover, by decreasing the amount of chondrocytes required (≤ 20% of the total cell mixture), culture-expansion is no longer necessary, which would allow the use of freshly isolated primary chondrocytes leading to improved cartilage formation. [33] Unfortunately, in depth understanding of the cellular interaction pathways between MSCs and chondrocytes is under debate in literature: It is thought that the co-culture effect is either credited by (1) chondrocyte driven MSC-differentiation or ascribed to (2) chondrocytes, whose cartilage-forming capacity and proliferation activity are enhanced in the presence of MSCs. [34] In recent years, the trophic and paracrine functions of MSCs appeared most critical in this process, rather than the simple chondrogenic differentiation of MSCs alone. However, little is known as to whether their trophic function is a general characteristic of MSCs or dependent on the origin of the MSC source. MSCs from several anatomical locations have been applied in co-culture. Independent on their origin, mixed cell cultures of chondrocytes and MSCs have been demonstrated to generally improve chondrogenesis as well as to reduce hypertrophy and tissue mineralization. [34–36] In contrast, three co-culture studies using adipose-tissue-derived MSCs (AMSCs) showed limited or decreased effects of MSCs on chondrogenesis. [37–39] Such effect was hardly seen in co-culture studies using bone-marrow-derived MSCs (BMSCs), which may propose that, compared to BMSCs, AMSCs are less efficient in co-culture. Due to methodological heterogeneity however, a direct comparable analysis between AMSCs and BMSCs in co-culture could not be easily made. So far, only three research groups have directly compared the effect of AMSCs and BMSCs on chondrocytes in co-culture. [40–42] Unfortunately, these studies demonstrate conflicting outcomes and have never translated to animal research.

Therefore, we aim to investigate whether MSCs undergo chondrogenic differentiation upon contact with chondrocytes or by trophic effects of MSCs on chondrocytes. Whether the co-culture effect is dependent on MSC-origin or a general characteristic of MSCs, is further elucidated. Therefore, chondrogenesis of human AMSCs (*h*AMSCs) and BMSCs (*h*BMSCs) combined with bovine articular chondrocytes (*b*ACs) is compared. The xenogeneic set-up using *h*MSCs and *b*ACs will allow conclusions about the cell type responsible for chondrogenesis. As cellular interactions can be influenced or overruled by exogenous growth factors, no growth factors are added to the culture system to study cartilage formation of the co-cultures *in vitro*. Moreover, cartilage formation will be evaluated after immediate subcutaneous implantation of the constructs in mice. To further elucidate the interactions between MSCs and ACs, different *in-vitro* culture systems will be used: (1) co-culture system of *h*MSC/*b*ACs in alginate, (2) pellet co-culture system of *h*MSC/*b*ACs, (3) Transwell® system of singular isolated *h*MSCs and *b*ACs in alginate, and (4) conditioned media culture systems of conditioned medium of *h*MSCs on *b*ACs and vice versa.

## Materials and methods

Chemicals were obtained from Sigma-Aldrich, USA unless stated otherwise.

### Cell sources

All human samples were obtained after approval by the Erasmus MC Medical Ethical Committee. Human mesenchymal stem cells (*h*MSCs) were isolated from either adipose tissue (*h*AMSCs) or bone-marrow aspirates (*h*BMSCs). *h*AMSCs were obtained from subcutaneous abdominal adipose tissue as waste material without the need for informed consent (protocol # MEC-2011-371) (*n = 3 independent donors*: F 52Y; F 51Y; F 53Y). *h*BMSCs were isolated from bone-marrow heparinized aspirates, after written informed consent had been acquired (protocol # MEC-2004-142 and Albert Schweitzer Hospital 2011/7) (*n = 3 independent donors*: M 67Y; F 75Y; M 22Y). Both *h*AMSCs and *h*BMSCs were seeded and cultured overnight in medium consisting of Minimum Essential Medium Alpha (MEM-α; Gibco, USA), supplemented with 10% fetal calf serum (FCS; Lonza, the Netherlands), 10^−4^ M L-ascorbic acid 2-phosphate, and 1 ng/mL basic Fibroblast Growth Factor 2 (bFGF2; AbD Serotec, UK). [43–45]

Articular chondrocytes (ACs) were selected, to study the trophic effect of *h*AMSCs or *h*BMSCs on chondrocytes. To obtain primary bovine articular chondrocytes (*b*ACs), macroscopically intact cartilage was harvested from the metatarsophalangeal joints of calves ≤ 6 months old (T. Boer & Zn., Nieuwerkerk aan den IJssel, the Netherlands), and washed with saline (*n = 4* pools of 3 donors each). To isolate cells, cartilage pieces were incubated for 1 hour with 2 mg/mL protease (type XIV derived from Streptomyces griseus), followed by overnight incubation with 1.5 mg/mL collagenase B (Roch Diagnostics, Germany) in High Glucose—Dulbecco's Modified Eagle's Medium (HG-DMEM; Gibco) with 10% FCS, 50 μg/mL gentamycin (Gibco), and 0.5 μg/mL amphotericin B (Fungizone; Life Technologies, Breda, the Netherlands). To extract small parts of undigested cartilage, the cell suspension was filtered through a nylon 100-μm mesh. Prior to cell culture, cell viability was tested using the trypan blue exclusion test, and cell number was calculated with a hemocytometer.

### Chondrogenesis

For *in-vitro* and *in-vivo* studies, all cells were encapsulated in alginate (Batch MG-004, CellMed, Germany), a hydrogel known of its high biocompatibility [46] and chondrogenic capacity [47]. Moreover, alginate hydrogels enable homogeneous cell distribution and allow paracrine factors to access all cells equally [47], making them suitable scaffolds for following research purposes.

Second-passaged *h*MSCs and non-expanded primary *b*ACs were harvested and cultured in a 3D-alginate hydrogel. Cells were suspended at a density of 4x10^6^ cells/mL in clinical grade 1.1% low viscosity alginate solution dissolved in 0.9% NaCl as single-cell-type populations or as a combination of 80% *h*MSCs (either *h*AMSCs or *h*BMSCs) and 20% *b*ACs. (Table 1) A 4:1 ratio was selected based on our previous experience [48] and that of others [49, 50].

**Table 1:** Construct conditions.

	Human stem cells		Bovine chondrocytes	
	Source	Cell density (x10^6^)	Source	Cell density (x10^6^)
***h*AMSC**	*h*AMSCs	4 nc/mL	x	x
***h*BMSC**	*h*BMSCs	4 nc/mL	x	x
***b*AC**	x	x	*b*ACs	4 nc/mL
***h*AMSC/*b*AC**	*h*AMSCs	3.2 nc/mL	*b*ACs	0.8 nc/mL
***h*BMSC/*b*AC**	*h*BMSCs	3.2 nc/mL	*b*ACs	0.8 nc/mL
**Control *b*AC**	x	x	*b*ACs	0.8 nc/mL

Cell density is displayed as the number of cells (nc) in 1 milliliter of alginate. *h*AMSC = human Adipose-tissue-derived Mesenchymal Stem Cell; *h*BMSC = human Bone-marrow-derived Mesenchymal Stem Cell; *b*AC = bovine Articular Chondrocyte.

**Fig 1:**
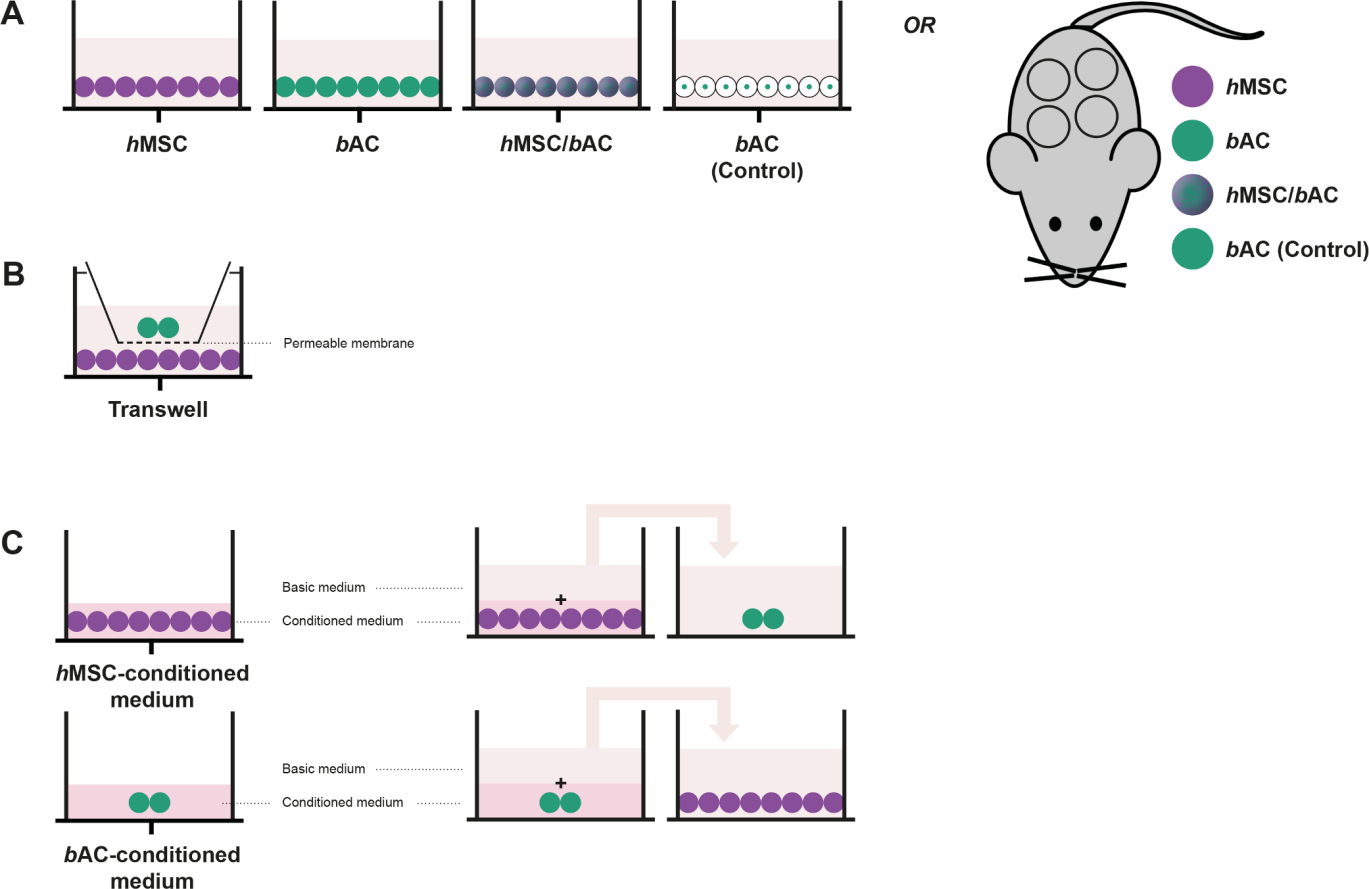
Cellular interaction. Cells were encapsulated in alginate beads separately and alginate and pellet co-cultures **(A, control conditions)**. Furthermore, *h*MSCs and *b*ACs were co-cultured in **(B)** a Transwell® system as well as in **(C)** medium conditioned by the other cell type, to further understand the complex cellular communication pathways between *h*MSCs and *b*ACs. In purple: *h*MSCs = human Mesenchymal Stem Cells; in green: *b*ACs = bovine Articular Chondrocytes.

Flat constructs (8 mm diameter; 2 mm height) were processed as previously described. [2] In short, alginate suspensions were injected into a custom designed slab mold consisting of 2 calcium-permeable membranes (Durapore® 5.0 μm membrane filters, Millipore) rigidly supported by stainless-steel meshes and separated by a stainless-steel casting frame. Alginate was instantaneously gelated for 30 minutes in 102 mM CaCl_2_ and thereafter washed with 0.9% NaCl and HG-DMEM. Sterile biopsy punches (Spengler, Asnières sur Seine, France) were used to create alginate constructs suitable for mechanical testing. Constructs were either cultured *in vitro* or directly implanted subcutaneously in mice. (Fig 1A)

*In vitro*, constructs were cultured in ‘basic medium’ containing serum-free HG-DMEM supplemented with 50 μg/mL gentamycin; 0.5 μg/mL Fungizone; 1 mM sodium pyruvate (Gibco); 40 μg/mL L-proline; supplemented Insulin Transferrine Selenium (ITS+; B&D Bioscience, Bedford, MA, USA); 10^−7^ M dexamethason; and 25 μg/mL L-ascorbic acid 2-phosphate without the addition of growth factors. For each condition referred to in Table 1, 3 independent donors were used in triplicate (total *n = 54*). After 3 and 5 weeks, constructs were processed for biochemical and gene-expression analysis.

*In-vivo* studies were completed after 8 weeks of subcutaneous implantation. In total, 10 9-week-old, female NMRI nu/nu mice (Charles River Laboratories, the Netherlands) were used. Two separate incisions were made along the central line of the spine (1 at the shoulders and 1 at the hips), after which 4 separate subcutaneous dorsal pockets were prepared by blunt dissection. For each condition referred to in Table 1, 3 independent donors were used in duplicate (total *n = 36*). Moreover, cell-free constructs were used as controls (*n = 4*). For implantation, alginate constructs were randomly assigned to these 4 pockets. After 8 weeks, animals were sacrificed and samples were explanted for histological, biomechanical and biochemical analyses. Animal experiments were carried out to the guidelines prescribed by the Dutch National Institutes of Health, and were approved by the Dutch equivalent of the Institutional Animal Care and Use Committee, the Erasmus MC Dier Ethische Commissie (protocol # EMC 2429).

### Cellular interaction

To further understand the complex cellular communication pathways between MSCs and ACs, cell types (*h*AMSCs (F 53Y); *h*BMSCs (M 22Y); *b*ACs pool of 3 donors) were co-cultured as follows: (1) *h*MSCs and *b*ACs were combined and cultured in alginate as previously described; (2) *h*MSCs and *b*ACs were cultured in pellets, allowing direct cell-cell contact. Furthermore, *h*MSCs and *b*ACs were encapsulated in alginate separately and co-cultured in (3) a Transwell® system; as well as (4) in medium conditioned by the other cell type. (Fig 1) The ratio of *h*MSCs to *b*ACs in each culture system was kept 80:20 for all conditions. All constructs were cultured under standardized nutritional conditions. Medium was changed 3 times a week. After 3 weeks, alginate beads and pellets were processed for biochemical or gene-expression analysis.

#### (1) Co-culture

*h*MSCs and *b*ACs were suspended at a density of 4x10^6^ cells/mL in clinical grade alginate solution as a mixed-cell-type population at a 80:20 ratio as described above. (Fig 1A)

#### (2) Pellet culture

To study the effects of direct cell-cell contact in co-cultures, *h*MSCs and *b*ACs were cultured in pellets. Therefore, a mixture of 80% *h*MSCs and 20% *b*ACs was suspended in basic medium and a total number of 2,5x10^5^ cells in 0.5 mL were transferred into polypropylene tubes and pellet were formed by centrifuging at 200 G for 8 minutes. To induce proper pellet formation, addition of Transforming Growth Factor β1 (TGFβ1; R&D Systems, USA) for 24 hours was required. This exposure was not sufficient to induce chondrogenesis in *h*MSCs (data not shown). After 24 hours, pellet were exposed to the ‘basis medium’ without addition of any growth factors. (Fig 1A)

#### (3) Transwell® system

*h*MSCs and *b*ACs were suspended at a density of 4x10^6^ cells/mL in clinical grade alginate solution as single-cell-type populations and transferred into a 10-mL sterile syringe. Thereafter, the cell-suspension was slowly passed through a 23-gauge needle to produce drops, which fell into a 102 mM CaCl_2_ creating alginate beads. Following instantaneous gelation, beads were allowed to further gelate for a period of 10 minutes in the CaCl_2_-solution. After being washed once with 0.9% NaCl and HG-DMEM, the beads were transferred to a Transwell® system (Corning Life Science, USA). The Transwell® inserts separated *h*MSCs and *b*ACs by a porous membrane of 8 μm, allowing paracrine signaling between *h*MSCs and *b*ACs. (Fig 1B)

#### (4) Conditioned medium

Alginate beads containing *h*MSCs or *b*ACs were produced as described above and cultured in medium conditioned by the other cell types. To obtain *b*ACs, *h*AMSCs and *h*BMSCs conditioned media, alginate beads were cultured in ‘basic medium’ for 3 days. After 3 days of culture, conditioned media were collected, enriched with 1:1 ‘basic medium’ and immediately added to alginate cultures of the other cell types. Again, a 80:20 ratio between *h*MSCs and *b*ACs was maintained. (Fig 1C)

### Biochemical evaluation of the extracellular matrix

Alginate constructs were digested overnight at 56°C in papain (250 μg/mL in 0.2 M NaH2PO4, 0.01 M EDTA, containing 5 mM L-cystein; pH 6.0); pellets were digested overnight at 56°C in proteinase K (1 mg/mL in Tris/EDTA buffer containing 185 μg/mL iodoacetamide and 1 μg/mL pepstatin A; pH 7.6). After digestion, samples were subjected to biochemical analyses to determine DNA, glycosaminoglycan (GAG), and hydroxyproline contents as described previously. [2] In short, the amount of DNA was determined by Ethidium bromide (GibcoBR1), using calf thymus DNA as a standard. Sulphated GAGs were quantified by the 1,9-Dimethylmethylene blue (DMMB) dye-binding assay, using shark chondroitin sulphate C as a standard. To be suitable for cell cultures containing alginate, the DMMB-pH-level was adjusted to pH 1.75, as described previously. [51] For the hydroxyproline content, digests were hydrolysed, dried and redissolved in 150 μL water. Hydroxyproline contents were measured using chloramine-T and dimethylaminobenzaldehyde as reagents and hydroxyproline (Merck, Germany) as a standard. Collagen content was subsequently estimated from the hydroxyproline content, assuming that one collagen triple helix molecule contains 300 hydroxyproline residues.

### Histological evaluation

After 8 weeks of subcutaneous implantation, constructs were harvested, set in 2% agarose, fixed in 4% formalin in PBS and embedded in paraffin. Paraffin-embedded sections (6 μm) were deparaffinised and rehydrated.

To evaluate tissue calcification, Von Kossa staining was performed. Slides were immersed in 5% silver nitrate solution for 10 minutes, rinsed in MilliQ and exposed to light for another 10 minutes. Excess silver nitrate was removed with 5% sodium-thiosulphate and slides were rinsed in distilled water afterwards. Sections were counterstained with Nuclear fast red (Merck).

To examine proteoglycans present in the newly synthesized ECM, deparaffinised sections were stained with Safranin-O and fast green.

To allow the use of monoclonal mouse antibody collagen type II (II-II6B3 1:100; Developmental Studies Hybridoma Bank, USA) on constructs which had been implanted in mice, the primary antibody was pre-coupled overnight with goat anti-mouse biotin at 4°C (1:500; Jackson Laboratories, USA), followed by a 2-hour incubation in 0.1% normal mouse serum (CLB, the Netherlands), to prevent unwanted binding of the anti-mouse antibodies to mouse immunoglobulins. [52] Antigen retrieval was performed through incubation with 0.1% pronase for 30 minutes at 37°C, continued with a 30 minutes incubation with 1% hyaluronidase at 37°C. Non-specific binding sites were blocked with 10% goat serum and sections were stained with the pre-treated antibodies for 60 minutes. Sections were than incubated with enzyme-streptavidin conjugate (Label, 1:100, Biogenex, HK-321-UK, USA) in PBS/1% BSA, followed by incubation with Neu Fuchsin substrate (Chroma, Germany).

### Biomechanical analysis

In order to distinguish the mechanical strength of alginate itself, cell containing constructs were prepared and directly taken for mechanical testing as described previously. [2] In short, for mechanical characterization of engineered cartilage constructs after *in vivo* cell culture, constructs 2.5 mm thick and 5 mm in diameter were used. The samples were placed in close-fitting Ø 5 mm stainless steel cylindrical wells. Mechanical testing was performed with a materials testing machine (Zwick Z005, Ulm, Germany) equipped with a 10 N load cell, a built-in displacement control, and a cylindrical, plane ended, stainless steel indenter (Ø 1.2 mm). During mechanical testing the samples were immersed in PBS. Stress-strain testing was performed: the samples were compressed to a final height of 0.5 mm at a loading rate of 5 mm per minute. An in-house Matlab® script was used to locate the sample surface and measure the sample thickness. Force-displacement curves were then converted to stress-strain curves. Measurements of compressive modulus at 40% strain, E40%, were determined for every sample.

### Gene-expression analyses

For total RNA isolation, alginate was dissolved in ice-cold 55 mM sodium citrate and 20 mM Ethylene Diamintetraacetate (EDTA) in 150 mM NaCl and centrifuged. Each cell-pellet was subsequently suspended in 1 mL RNA-Bee^TM^ (TEL-TEST, USA). For total RNA isolation from pellets, pellets were manually homogenized and suspended in 300 μL/pellet RNA-Bee^TM^. RNA was extracted with chloroform and purified from the supernatant using the RNAeasy Micro Kit (Qiagen, Germany) according to the manufacturer’s guidelines by on-column DNA-digestion. Extracted total RNA was quantified using NanoDrop® ND-1000 Spectrophotometer (NanoDrop Technologies, Wilmington, DE, USA) at 260/280 nm. Total RNA of each sample was reverse transcribed into cDNA using RevertAidTM First Strand cDNA Synthesis Kit (MBI Fermentas, Germany).

For quantitative real-time Polymerase Chain Reaction (qRT-PCR) analysis, forward and reverse primers were designed using PrimerExpress 2.0 software (Applied Biosystems, USA) to meet TaqMan or SYBR Green requirements. Gene specificity of all primers was guaranteed by Basic Local Alignment Search Tool (BLASTN). Analysed genes are listed in Table 2. qRT-PCR was performed using qPCR Mastermix Plus for SYBR Green (Eurogentec, the Netherlands) according to the manufacturers’ guidelines and using ABIPRISM® 7000 with SDS software version 1.7 (Applied Biosystems, the Netherlands). Relative gene expressions were calculated by means of the 2^-ΔCT^ formula.

**Table 2:** Sequences of primers for qRT-PCR.

Primers and probes	
**Human specific genes**	
***hsGAPDH***	Fw: AGCTCACTGGCATGGCCTTC
	Rev: CGCCTGCTTCACCACCTTCT
***hsACAN***	Fw: CAGCCACCACCTACAAACGCAG
	Rev: CTGGGTGGGATGCACGTCAGC
***hsCOL2A1***	Fw: ACGAGGCCTGACAGGTCCCA
	Rev: GCCCAGCAAATCCCGCTGGT
**Bovine specific genes**	
***bsGAPDH***	Fw: GTCAACGGATTTGGTCGTATTGGG
	Rev: TGCCATGGGTGGAATCATATTGG
***bsACAN***	Fw: GGACACTCCTTGCAATTTGAGAA
	Rev: CAGGGCATTGATCTCGTATCG
***COL2A1***	Fw: GGCAATAGCAGGTTCACGTACA
	Rev: CGATAACAGTCTTGCCCCACTT

*GAPDH* = GlycerAldehyde 3-Phosphate DeHydrogenase; *ACAN* = Aggrecan; *COL2A1* = Collagen type 2; *hs* = human-specific; *bs* = bovine-specific.

### Statistical analysis

All data were analyzed with PSAW statistics 20.0 (SPSS inc. Chicago, USA). For *in vitro* alginate co-cultures, the mean and standard deviation represents at least three independent donors per cell source performed in triplicate. For statistical evaluation, a mixed linear model was used followed by a Bonferroni's post-hoc comparisons test. Condition and time point were defined as fixed factors in the model. Donor and sample number were treated as random factors. For *in vivo* alginate co-cultures, the mean and standard deviation represents at least three independent donors per cell source performed in duplicate. For the evaluation of the cellular communication pathways between MSCs and ACs, the mean and standard deviation represents one donor per cell source performed in sextuple. For statistical evaluation, the Kruskal-Wallis followed by the Mann-Whitney-U tests was used followed by a Bonferroni's post-hoc comparisons test. For all tests, values of *p*<0.05 were considered statistically significant.

## Results

### Cartilage regeneration in co-cultures

#### *In vitro* outcomes

**Fig 2:**
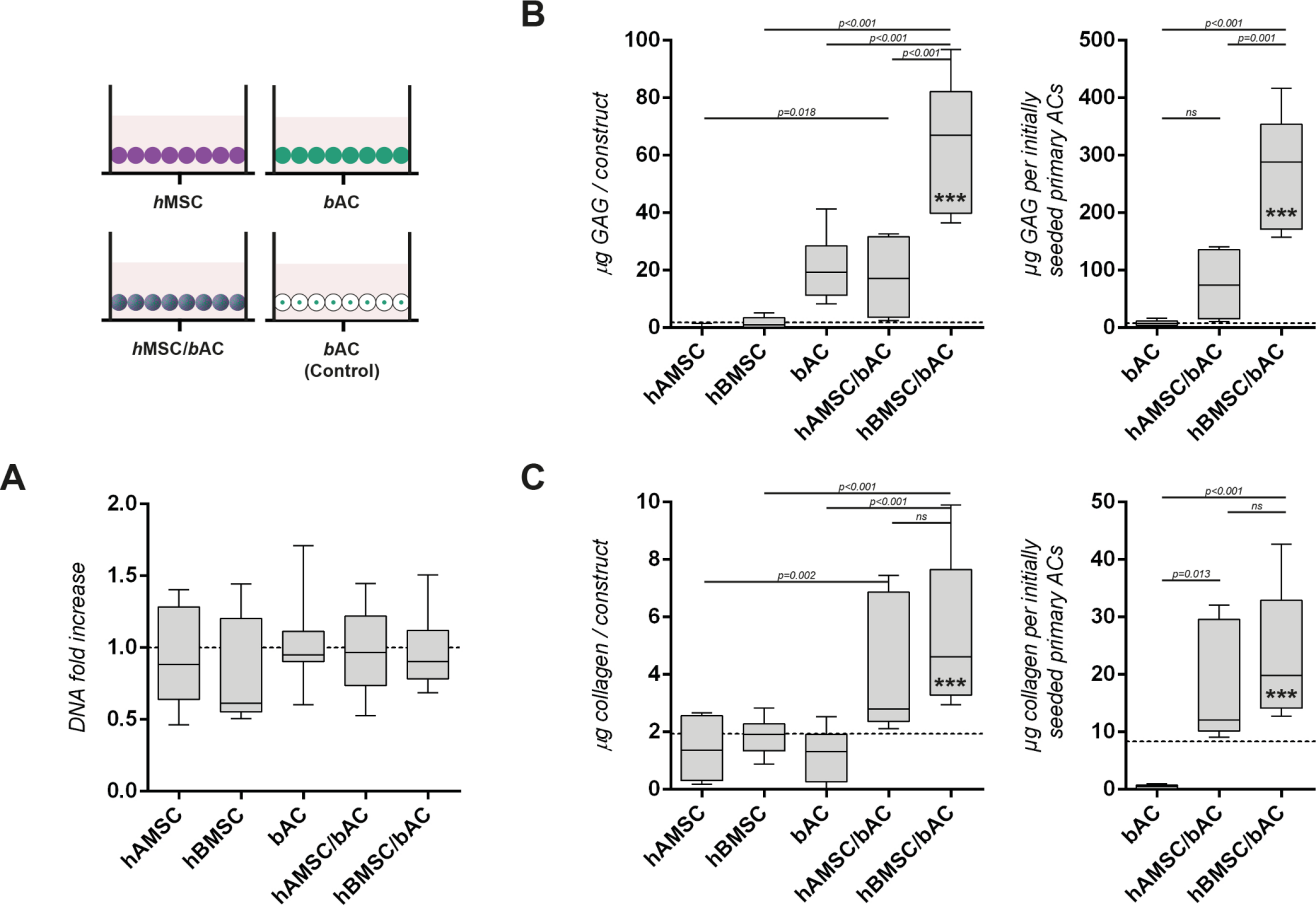
Cartilage matrix formation in constructs containing *h*MSCs and/or *b*ACs, 3 weeks after *in-vitro* culture. **(A)** The DNA content of none of the constructs had changed compared to their initial DNA content prior to cell-culture (dotted line). Biochemical evaluation of the GAG **(B)** and collagen **(C)** content, 3 weeks after culture in alginate. The left graphs demonstrate the amount of matrix components per construct, whereas for the right graphs matrix production is normalized to the initially seeded primary ACs. A control condition—containing similar amounts of *b*ACs (0.8*10^6^ nc/ml) without supplementation of *h*MSCs—was evaluated to determine the additional effect of *h*MSCs (3.2*10^6^ nc/ml) on *b*ACs in co-cultures (dotted line). *, ** or *** indicates p-values smaller than 0.05, 0.01 or 0.001 respectively compared to the control condition. Data are shown as mean ± SD. For statistical evaluation, a mixed model was used followed by a Bonferroni's post-hoc comparisons test. *h*AMSC = human Adipose-tissue-derived Mesenchymal Stem Cell (*n = 3* experiments with 3 independent donors); *h*BMSC = human Bone-marrow-derived Mesenchymal Stem Cell (*n = 3* experiments with 3 independent donors); *b*AC = bovine Articular Chondrocyte (*n = 3* experiments with 3 pools of donors). Per experiment, 3 samples were used for analyses.

After 3 weeks, DNA content of alginate constructs containing either co-cultures of *h*MSCs and *b*ACs or single-cell-type populations, did not change in relation to their initial DNA content. (Fig 2A) Because the amount of DNA had not changed significantly in any of the conditions, matrix deposition was expressed per construct and per initially seeded primary ACs. After 5 weeks, DNA content did significantly decrease in constructs containing *h*BMSCs only (*p*<0.001), but remained unchanged in the remaining culture conditions. (S1 Fig)

Since constructs were cultured in the absence of chondrogenic factors, constructs containing solely *h*AMSCs or *h*BMSCs produced very little GAG (Fig 2B) and collagen (Fig 2C). To demonstrate the additional effect of *h*MSCs in mixed-cell-type populations, a control condition—containing similar numbers of *b*ACs (0.8*10^6^ nc/mL) without the supplementation of *h*MSCs—was evaluated (Fig 2 dotted lines). The addition of either *h*AMSCs or *h*BMSCs to *b*ACs demonstrated a significant increase in the production of GAG over their controls (*h*AMSC/*b*ACs *p* = 0.018; *h*BMSC/*b*ACs *p*<0.001). Compared to constructs containing single-cell-type populations, the deposition of GAG was most evidently enhanced in co-cultures combining *h*BMSCs and *b*ACs (*p*<0.001). Constructs containing *h*AMSC/*b*ACs deposited significantly less GAG compared to *h*BMSC/*b*ACs (*p*<0.001) and equal amounts compared to constructs containing *b*ACs only. (Fig 2B) The production of collagen was enhanced in co-cultures of both *h*AMSC/*b*ACs and *h*BMSC/*b*ACs compared to single-cell-type populations (*h*AMSC/*b*ACs *p* = 0.002; *h*BMSC/*b*ACs *p*<0.001). (Fig 2C) Normalization of the total GAG content to the initially seeded primary ACs revealed even more distinct differences between co-cultures and single-cell-type populations: *h*BMSC/*b*ACs produced significantly more GAG compared to *b*ACs only and co-cultures of *h*AMSC/*b*ACs (both *p*<0.001); collagen production was significantly enhanced in both co-cultures (*h*AMSC/*b*ACs *p* = 0.013; *h*BMSC/*b*ACs *p*<0.001). (Fig 2B and 2C) Similar results were obtained after 5 weeks of culture. (Data not shown) These results demonstrate that co-cultures of *h*MSCs and *b*ACs improve cartilage formation *in vitro*, depending on the *h*MSC-source used (*h*BMSC ≥ *h*AMSC).

#### *In vivo* outcomes

**Fig 3:**
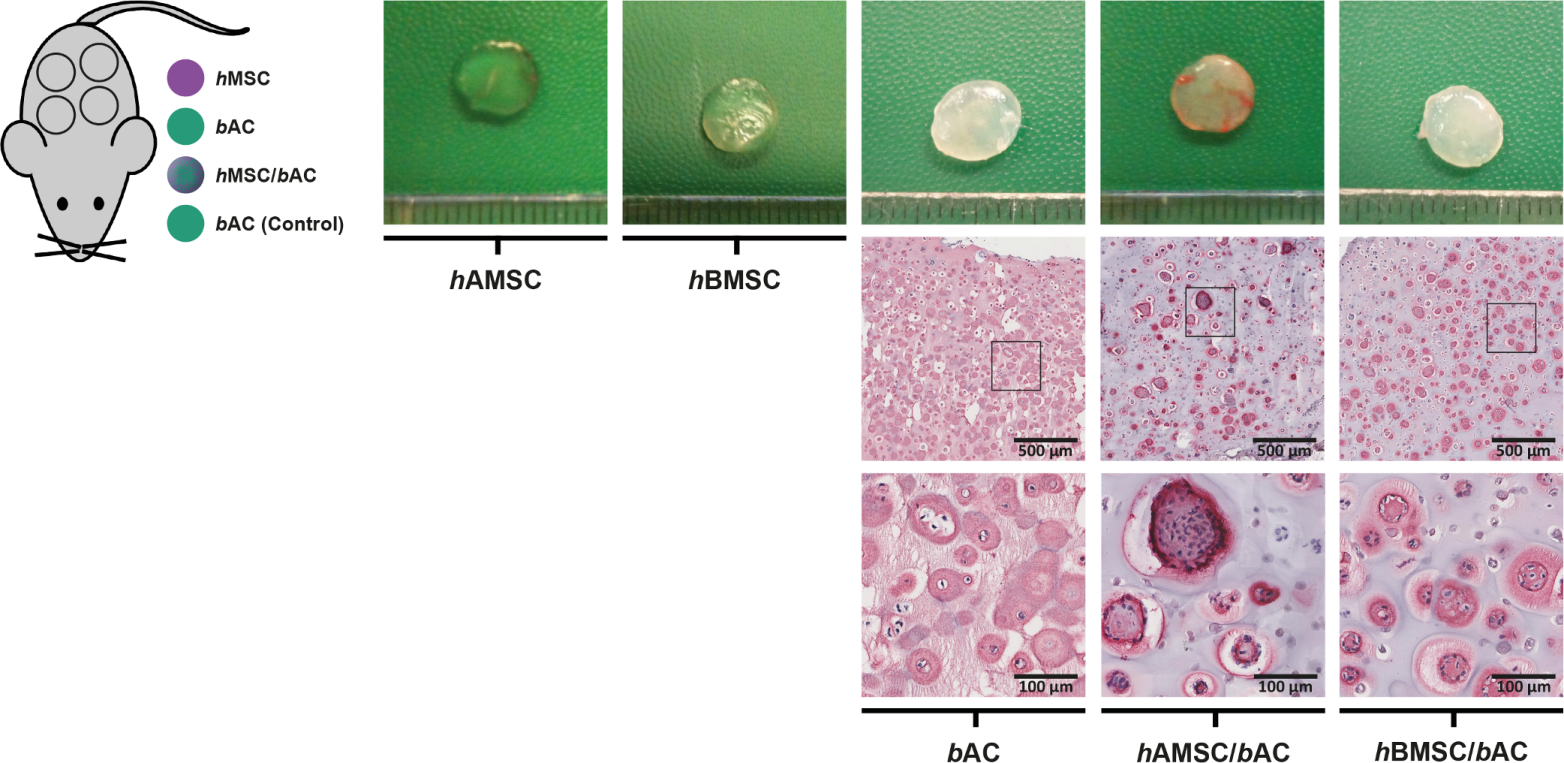
Macroscopic appearance and immunohistochemical analyses of constructs containing *h*MSCs and/or *b*ACs, 8 weeks after subcutaneous implantation in mice. Macroscopic appearance (top row) of cartilage constructs, as well as a collagen type II immunohistochemical staining (bottom rows), 8 weeks after subcutaneous implantation. *h*AMSC = human Adipose-tissue-derived Mesenchymal Stem Cell (*n = 3* experiments with 3 independent donors); *h*BMSC = human Bone-marrow-derived Mesenchymal Stem Cell (*n = 3* experiments with 3 independent donors); *b*AC = bovine Articular Chondrocyte (*n = 3* experiments with 3 pools of donors). Per experiment, 2 samples were used for analyses.

Cell-free alginate constructs (controls; *n = 4*) and alginate constructs containing *h*BMSC/*b*ACs, *h*AMSC/*b*ACs or *h*BMSC, *h*AMSC or *b*AC only, were generated and immediately implanted subcutaneously in athymic mice. After 8 weeks, all but 5 (*n = 3 h*AMSC, *n = 2 h*BMSC) of the 40 constructs could be identified and harvested. Unfortunately however, the remaining *h*MSC-constructs (either *h*AMSCs or *h*BMSCs) and cell-free alginate constructs were lost during the embedding process. Constructs containing *b*ACs or *h*BMSC/*b*ACs resembled cartilage tissue in both color and texture, while the appearance of constructs containing *h*AMSC/*b*ACs was particularly donor-dependent. (Fig 3) None of the constructs had mineralized or ossified. Also, vascularization within the construct, was never observed. Cells were more heterogeneously distributed in constructs containing either *h*AMSC/*b*ACs or *h*BMSC/*b*ACs compared to *b*ACs only. Collagen type II was abundantly present in constructs containing *b*AC or *h*BMSC/*b*ACs. Again, hAMSC/bACs contained collagen type II in a donor-dependent manner. (Fig 3) A Safranin-O staining displayed similar results. (S2 Fig)

**Fig 4:**
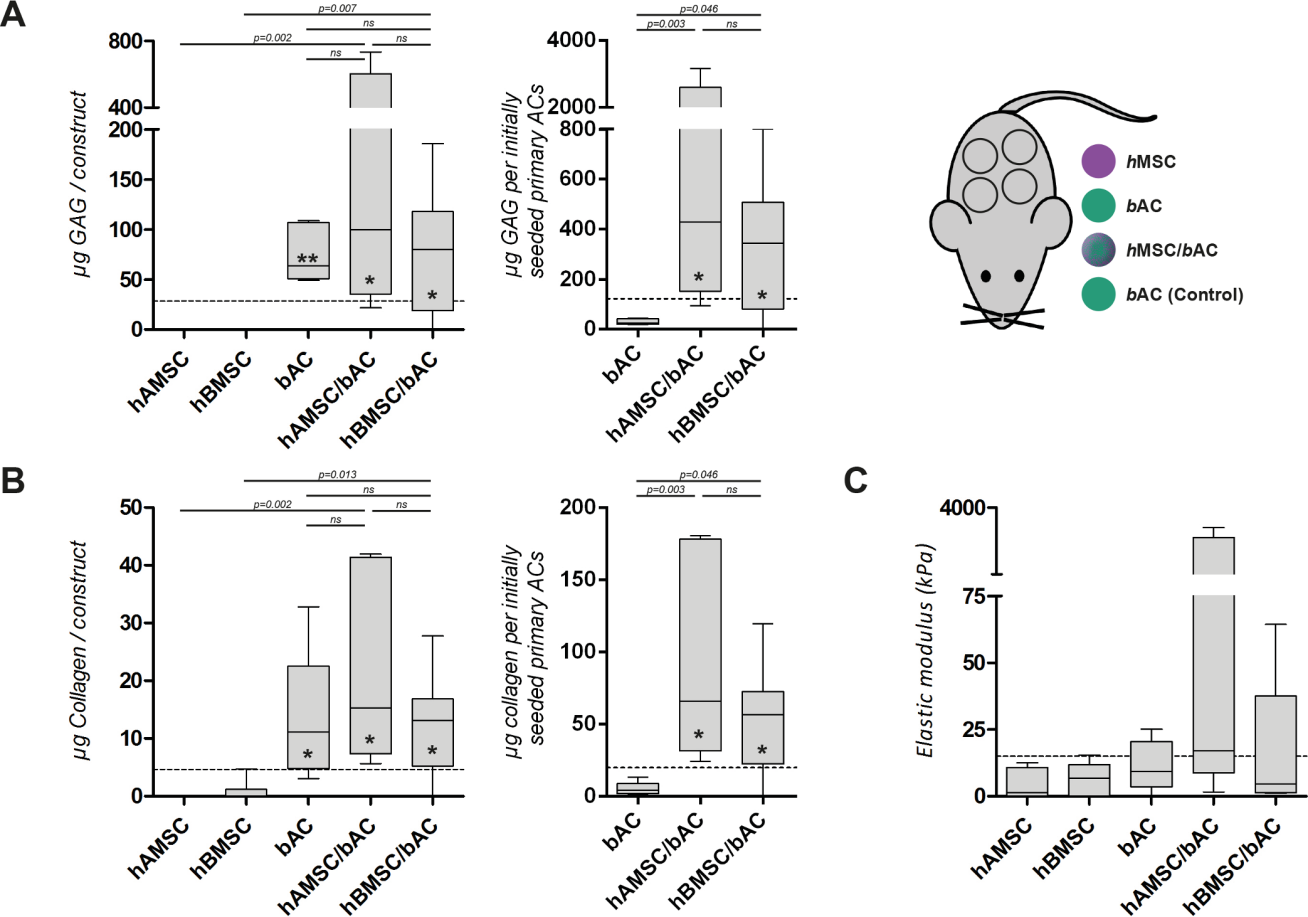
Cartilage matrix formation in constructs containing *h*MSCs and/or *b*ACs, 8 weeks after subcutaneous implantation in mice. Biochemical (GAG **(A)** and collagen **(B)** content) and biomechanical evaluation **(C)**, 8 weeks after subcutaneous implantation. The left graphs in A and B, demonstrate the amount of matrix components per construct, whereas for the right graphs matrix production is normalized to the initially seeded primary ACs. A control condition—containing similar amounts of *b*ACs (0.8*10^6^ nc /ml) without supplementation of *h*MSCs—was evaluated to determine the additional effect of *h*MSCs (3.2*10^6^ nc /ml) on *b*ACs in co-cultures (dotted line). *, ** or *** indicates p-values smaller than 0.05, 0.01 or 0.001 respectively compared to the control condition. Data are shown as box-whisker plots. For statistical evaluation, a Kruskal-Wallis followed by the Mann-Whitney-U test was used followed by a Bonferroni's post-hoc comparisons test. *h*AMSC = human Adipose-tissue-derived Mesenchymal Stem Cell (*n = 3* experiments with 3 independent donors); *h*BMSC = human Bone-marrow-derived Mesenchymal Stem Cell (*n = 3* experiments with 3 independent donors); *b*AC = bovine Articular Chondrocyte (*n = 3* experiments with 3 pools of donors). Per experiment, 2 samples were used for analyses.

*In vivo*, DNA- and GAG-content were not detected in cell-free alginate constructs. (Data not shown) *h*AMSC/*b*ACs and *h*BMSC/*b*ACs contained similar quantities of cartilage matrix as constructs containing *b*ACs only. Moreover GAG formation in co-cultures was independent of the origin of the *h*MSC-source used (*p* = 0.916). (Fig 4A) Collagen production demonstrated a similar trend, again without statistical significant differences between *h*AMSC/*b*ACs and *h*BMSC/*b*ACs (*p* = 1.000). (Fig 4B) Normalization of the data to their initially seeded primary ACs revealed more distinct differences between mixed-cell-type and single-cell-type populations: *h*AMSC/*b*ACs and *h*BMSC/*b*ACs produced significantly more GAG and collagen per initially seeded primary ACs compared to *b*ACs (*h*AMSC/*b*ACs *p*<0.01; *h*BMSC/*b*ACs *p*<0.05). (Fig 4) After subcutaneous implantation, the elastic modulus was highest in constructs containing *h*AMSC/*b*ACs and *h*BMSC/*b*ACs, albeit this did not reach statistical significance due to the large variation between samples. (Fig 4C) These results confirm our *in-vitro* results by showing that co-cultures of *h*MSCs and *b*ACs improve cartilage formation. However, *in vivo* this phenomenon seems independent of the *h*MSC-source used, although large donor variation is observed.

### Differentiation versus chondroinduction

Using a xenogeneic *in-vitro* culture system enabled us to determine the contribution of each individual cell type (i.e. *h*BMSCs, *h*BMSCs or *b*ACs) to cartilage matrix production using species-specific gene-expression analyses.

**Fig 5:**
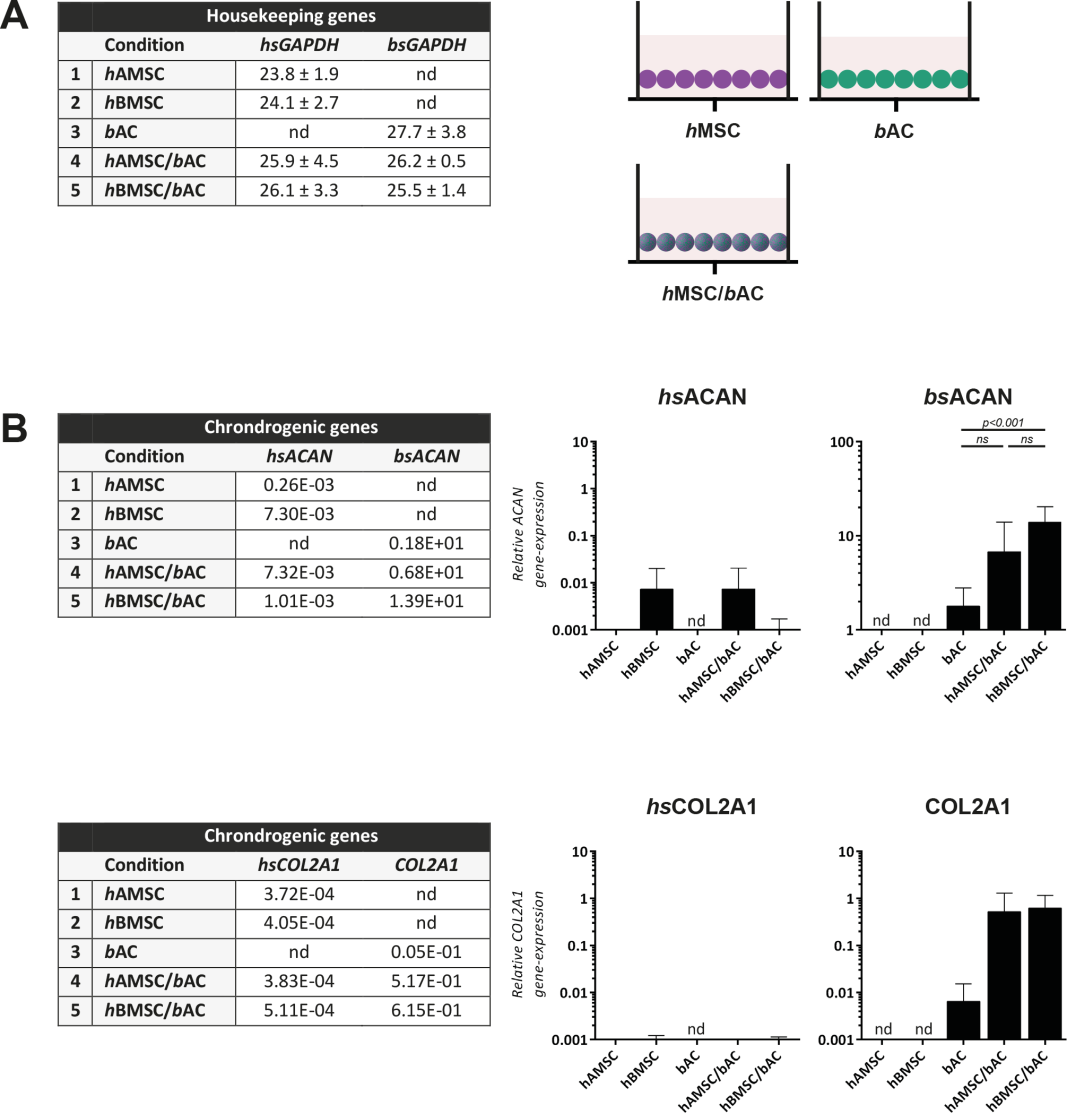
Gene-expression analysis, 5 weeks after *in vitro* culture. Data are shown as mean CT-values ± SD of housekeeping genes **(A)** and average relative gene-expression of chondrogenic genes **(B)**. nd = not detected (ct-value > 35.00); *hsGAPDH* = human-specific *GAPDH*; *bsGAPDH* = bovine-specific *GAPDH*; *hsACAN* = human-specific *ACAN*; *bsACAN* = bovine-specific *ACAN*; *hsCOL2A1* = human-specific COL2A1; *h*AMSC = human Adipose-tissue-derived Mesenchymal Stem Cell (*n = 3* experiments with 3 independent donors); *h*BMSC = human Bone-marrow-derived Mesenchymal Stem Cell (*n = 3* experiments with 3 independent donors); *b*AC = bovine Articular Chondrocyte (*n = 3* experiments with 3 pools of donors). Per experiment, 3 samples were used for analyses.

First, *GAPDH-*gene expression was analyzed after 5 weeks of *in-vitro* culture. *h*AMSC/*b*ACs and *h*BMSC/*b*ACs contained cells from both bovine (AC) and human (AMSC or BMSC) origin. (Fig 5A) Then, chondrogenic gene expression was evaluated by the *ACAN* and COL2A1 genes. In a growth-factor-free environment, *h*AMSCs and *h*BMSCs hardly expressed *hsACAN* and *hsCOL2A1*. Besides, *chondrogenic genes* were hardly expressed in *h*AMSC/*b*ACs or *h*BMSC/*b*ACs either. Conversely, *h*AMSC/*b*ACs or *h*BMSC/*b*ACs—containing solely 20% bovine articular chondrocytes—expressed as much or even higher levels of *bsACAN* compared to 100% *b*ACs (*h*AMSC/*b*ACs vs *b*ACs *p*>0.05; *h*BMSC/*b*ACs vs *b*ACs *p*<0.001). *h*AMSC/*b*ACs and *h*BMSC/*b*ACs expressed *COL2A1*, although gene-expression of *hsCOL2A1* was negligible. This means that the *COL2A1* expressed was from bovine origin. (Fig 5B) These data indicate that the formed cartilage matrix was from *b*AC-origin, which suggests a more trophic role for *h*MSCs herein.

### Cellular interactions

**Fig 6:**
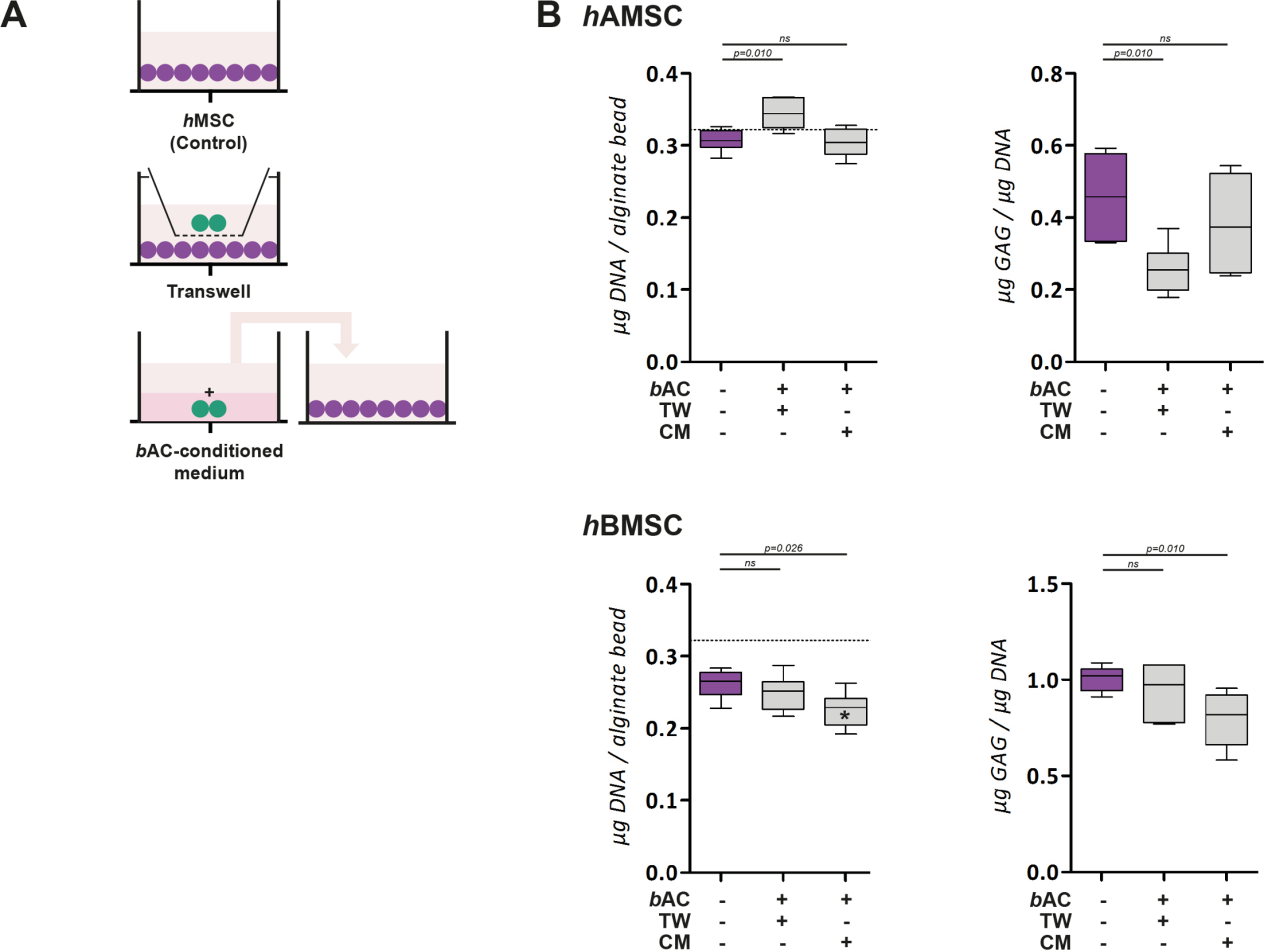
Paracrine effect of *b*ACs on *h*AMSCs and *h*BMSCs. **(A)** Schematic overview. In purple: *h*MSCs; in green: *b*ACs. **(B)** The DNA and GAG content of *h*AMSCs and *h*BMSCs in the presence of paracrine factors of *b*ACs via Transwell® system or *b*AC-conditioned medium. The DNA content after 3 weeks of culture was compared to the initial DNA content prior to cell-culture (dotted line). *, ** or *** indicates p-values smaller than 0.05, 0.01 or 0.001 respectively compared to the amount of DNA prior to cell culture. Data are shown as box-whisker plots of 6 samples of one experiment. For statistical evaluation, a Kruskal-Wallis followed by the Mann-Whitney-U test was use followed by a Bonferroni's post-hoc comparisons test. TW = Transwell; CM = Conditioned Medium; *h*AMSC = human Adipose-tissue-derived Mesenchymal Stem Cell; *h*BMSC = human Bone-marrow-derived Mesenchymal Stem Cell; *b*AC = bovine Articular Chondrocyte.

**Fig 7:**
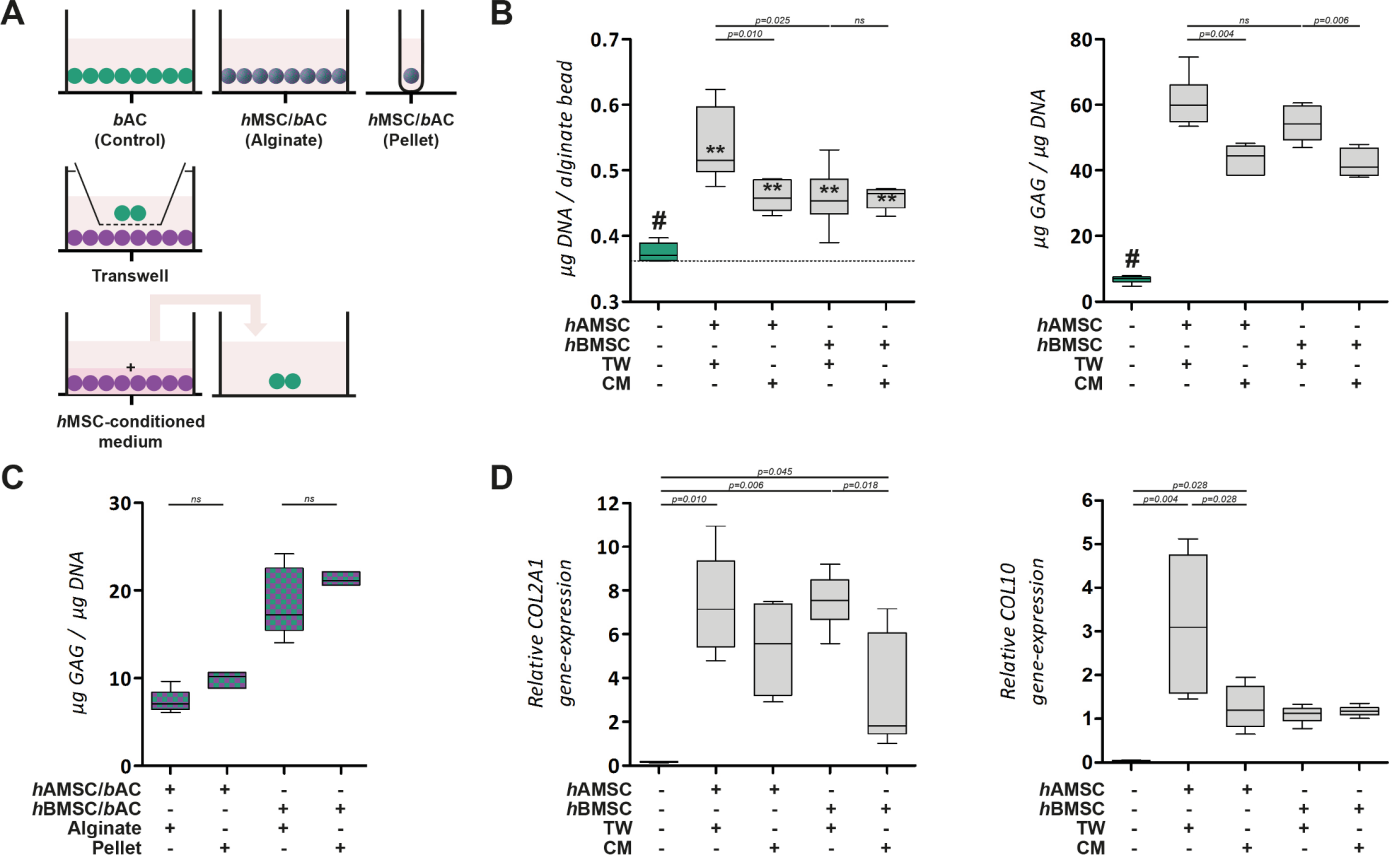
Paracrine effect of *h*AMSCs and *h*BMSCs on *b*ACs. **(A)** Schematic overview. In purple: *h*MSCs; in green: *b*ACs. **(B)** The DNA and GAG content of *b*ACs in the presence of paracrine factors of *h*MSCs via Transwell® system or *h*MSC-conditioned medium. The DNA content after 3 weeks of culture was compared to the initial DNA content prior to cell-culture (dotted line). **(C)** Co-culture in alginate constructs and pellet culture, allowing direct cell-cell contact. **(D)** Relative gene-expression analysis, 3 weeks after culture in alginate. *, ** or *** indicates p-values smaller than 0.05, 0.01 or 0.001 respectively compared to the amount of DNA prior to cell culture. # indicates significant differences from all conditions (*p*<0.01). Data are shown as box-whisker plots of 6 samples of one experiment. For statistical evaluation, a Kruskal-Wallis followed by the Mann-Whitney-U test was use followed by a Bonferroni's post-hoc comparisons test. TW = Transwell; CM = Conditioned Medium; *h*AMSC = human Adipose-tissue-derived Mesenchymal Stem Cell; *h*BMSC = human Bone-marrow-derived Mesenchymal Stem Cell; *b*AC = bovine Articular Chondrocyte.

To further understand the complex cellular interaction between *h*MSCs and *b*ACs, cells were encapsulated in separate alginate constructs and co-cultured in a Transwell® system as well as in medium conditioned by the other cell type. (Figs 6A and 7A) In addition cell combination were also cultured in pellets, allowing direct cell-cell contact.

Alginate constructs containing solely *b*ACs, *h*AMSCs or *h*BMSCs cultured in ‘basic medium’ maintained their DNA content over the 3 weeks of culture. Exposure to paracrine factors of *b*AC via Transwell® system or *b*AC-conditioned medium, did not alter the amount of DNA in alginate constructs seeded with either *h*AMSCs or *h*BMSCs. (Fig 6B) The presence of factors secreted by *h*MSC significantly increased the total amount of DNA in constructs containing *b*ACs (*p*<0.01). This effect was independent on the origin of the *h*MSCs (i.e. *h*AMSCs, *h*BMSCs) and co-culture system used (i.e. Transwell® system, *h*MSC-conditioned medium). (Fig 7B) This suggests MSC have paracrine effects on chondrocytes.

Alginate constructs containing *h*AMSCs or *h*BMSCs, formed very little GAG after 3 weeks of culture. GAG-production remained similarly low when *h*MSC-constructs were cultured in the presence of paracrine factors of *b*AC via Transwell® system or *b*AC-conditioned medium. (Fig 6B) The production of GAG was higher in constructs containing *b*ACs. Exposure to paracrine factors of *h*MSC significantly increased GAG-production, irrespective to the *h*MSC-source used (i.e. *h*AMSCs, *h*BMSCs, *p*<0.01). Since the amount of DNA was also enhanced in these constructs, GAG content was adjusted to the amount of DNA, still showing pronounced differences. GAG formation was significantly increased in Transwell® system compared to constructs cultured with *b*AC-conditioned medium (*p*<0.01). (Fig 7B) Similar trends were observed at *COL2A1* gene-expression level. (Fig 7D) This provides further indications that the effect of the combination of *h*MSCs and *b*ACs on chondrogenesis is due to paracrine effect of *h*MSCs on chondrocytes.

Based on previous results, we further wanted to evaluate signs of hypertrophy in these constructs, since hypertrophic differentiation is an unwanted phenomenon in cartilage regeneration. *b*ACs cultured in ‘basic medium’ expressed hardly any *COL10* after 3 weeks of culture. In addition, when *b*ACs were exposed to paracrine factors of *h*MSC either via Transwell® system or *h*MSC-conditioned medium, *COL10*-gene-expression was upregulated and significantly increased in constructs exposed to paracrine factors of *h*AMSCs (Transwell® system *p* = 0.004; *h*AMSC-conditioned medium *p* = 0.028). Although *COL10*-gene-expression was slightly upregulated in constructs exposed to paracrine factors of *h*BMSC, no significant differences could be observed in comparison with constructs cultured in ‘basic medium’ (both Transwell® system and *h*BMSC-conditioned medium *p*>0.05). (Fig 7D)

This indicates that *h*MSCs have the ability to improve cartilage matrix formation in co-culture, by improving *b*AC-proliferation capacity as well as increasing *b*AC-GAG-production. Moreover, when exposed to paracrine factors of *h*BMSC, hypertrophic differentiation was not significantly enhanced compared to untreated *b*ACs. In pellet co-culture, matrix production was similarly produced as in 3D-alginate constructs, meaning that direct cell-cell contact is not required for co-cultures of *h*MSCs and *b*ACs. (Fig 7C)

## Discussion

Combining chondrocytes and MSCs holds great promise for cell-based cartilage repair as it reduces the required number of chondrocytes and diminishes many disadvantages of individually used cell types leading to enhanced cartilage matrix formation with low hypertrophic differentiation. In line with former research, *h*AMSC/*b*ACs and *h*BMSC/*b*ACs produced similar or even improved quantities of cartilage matrix components as constructs containing *b*ACs only, both *in vitro* and *in vivo*. Moreover, hypertrophic gene expression (*COL10*) was not affected by *h*BMSCs, but slightly enhanced by *h*AMSCs. However, constructs containing either *h*AMSC/*b*ACs or *h*BMSC/*b*ACs appeared stable and did not calcify *in vivo*. This suggests that 80% of *b*ACs can be replaced by either *h*AMSCs or *h*BMSCs without influencing cartilage matrix production nor stability. Therefore, mixed-cell-cultures of MSCs and chondrocytes could be very valuable for cell-based cartilage repair, as appropriate numbers of cells are more easily acquired from bone-marrow aspirates or adipose tissue than from cartilage biopsies.

The cellular mechanism responsible for enhanced cartilage production in co-culture is however still debated. Numerous cellular communication pathways have been hypothesized in order to explain the beneficial effect in co-cultures [53]. We found no evidence that cartilage formation was the consequence of chondrogenic lineage differentiation of *h*MSCs, as stated by others [41, 49, 54–62]. In contrast, cartilage matrix clearly originated from *b*ACs, which suggests a predominantly trophic role for *h*MSCs in these constructs: both *h*AMSCs and *h*BMSCs improved *b*AC-proliferation as well as *b*AC-GAG-formation. This confirms previous studies were the co-culture effect has been ascribed to ACs, whose cartilage-forming capacity and proliferation activity appears to enhance in the presence of MSCs. [40, 50, 63–68] The trophic and paracrine function of MSCs herein appeared essential rather than MSCs actively undergoing chondrogenic differentiation. We show that this is a general feature that applies to both AMSCs and BMSCs.

To date, only three studies have compared the trophic effect of several MSC-sources—such as AMSCs and BMSCs—on ACs in co-culture. [40–42] Unfortunately, these studies demonstrate conflicting outcomes and have never translated to animal research. Therefore, to our knowledge, we are the first to systematically compare the cartilage forming capacity of either *h*AMSC/*b*ACs and *h*BMSC*/b*ACs *in vitro* and *in vivo*. *In vitro*, *h*BMSC/*b*ACs contained significantly more cartilage matrix components than *h*AMSC/*b*ACs. Cartilage formation after 8 weeks of subcutaneous implantation was, however, not different in constructs containing *h*AMSC/*b*ACs and *h*BMSC/*b*ACs, although large donor variations were observed, in particular in *h*AMSC/*b*ACs. Our results support a general trophic or immunomodulatory role for *h*AMSCs and *h*BMSCs on *b*ACs in co-culture, as stated by Wu [40] and Maumus *et al [42]*. Although both cell sources share comparable immunomodulatory modalities, they do not necessarily behave the same. In monocultures there are clear differences observed between *h*AMSCs and *h*BMSCs. For instance, they possess distinctive proliferation capacities and a dissimilar potential to chondrogenically differentiate. [2] Moreover, both cell sources secrete different subsets of paracrine factors: compared to *h*BMSCs, *h*AMSCs secrete significantly more VEGF-D [69], IGF-1 [69, 70], IL-8 [69] and IL-6 [69, 71], and significantly less SDF-1 [72] and TFGβ1 [72]. In co-cultures, differences between *h*MSC-cell sources appear less clear. Acharya *et al*. demonstrated enhanced chondrocyte proliferation capacity and improved GAG formation in pellets containing *h*BMSC/*b*ACs compared to *h*AMSC/*b*ACs. [41] Besides, 3 independent co-culture studies using AMSCs only showed limited or decreased effects of MSCs on chondrogenesis. [37–39] Such effect was hardly seen in co-culture studies using BMSCs only, which may propose that, compared to BMSCs, AMSCs seem less efficient in co-culture, Although we could not find a general beneficial effect of *h*BMSCs in co-cultures compared to *h*AMSCs *in vitro* and *in vivo*, we did show that *in vitro*, *h*BMSC/*b*ACs outperformed *h*AMSC/*b*ACs and hypertrophic gene expression was lower in *h*BMSC/bACs. True dissimilarities between *h*AMSCs and *h*BMSCs in co-culture are unfortunately hard to expose, as *h*MSC-cultures are highly heterogeneous and distinct population subsets will probably interfere with the reciprocal communication pathways in co-culture. Therefore, the purification of distinct subsets of *h*MSCs might enhance the particular capability of *h*AMSCs and *h*BMSCs in co-culture by eliminating interfering cells with limited potential, or even cells with inhibitory activity. Future research still needs to clarify whether the trophic role of MSCs in co-culture is truly a general MSC-characteristic produced by a distinct subset of the MSC-population or dependent on the original origin of the MSCs.

Our data and that of others emphasize the importance of paracrine signaling pathways in co-culture comparatively to juxtacrine or gap-junctional signaling. Although the importance of direct cell-cell contact is still unclear in literature [63], such signaling pathways remained less important in our study, since alginate hydrogel impedes direct cell-cell contact and in pellet culture no beneficial effect of direct cell-cell contact was observed. On the contrary, *b*ACs produced less cartilage matrix in Transwell® system with *h*MSCs and the amount of cartilage matrix was further reduced in *h*MSC-conditioned medium. Although direct cell-cell contact seems less significant than paracrine signaling, it seems correspondingly important to secure a certain cell-cell distance for optimal cell communication.

Furthermore, for optimal cell communication and subsequent cartilage regeneration, an optimal cell density and ratio of MSCs to ACs is imperative. Additionally, for cell-based cartilage repair, it would be ideal to only use low numbers of primary chondrocytes. Although Puelacher *et al* already recommended cell densities greater than 20x10^6^ cells per milliliter [73], we could not increase the cell seeding density over 4x10^6^ cells per milliliter, as the size of our experimental set-up did not enable higher densities. Additionally, we have replaced 80% of the *b*ACs by *h*MSCs (at a 4:1 ratio), as described previously. [40, 50] However, no consensus on optimal co-culture ratios is yet available. Future research needs to clarify if we could increase cell density while further reduce the number of primary chondrocytes (increase the MSC-chondrocyte-ratio) without inhibiting cartilage matrix production and stability.

The species mismatch limited the translation of presented basic research to clinical application. However, the species mismatch was chosen to be able to discriminate between the role of the different cell types. We do not expect huge differences in fully human co-culture models, as both xenogeneic and autologous co-culture models have resulted in comparable outcomes, indicating that in both models comparable mechanisms are likely operational. [50] Our results confirmed previously published results of *h*MSCs combined with xenogeneic chondrocytes. [50, 74–76] Therefore, it appears to be an excellent model to study cell-specific contributions to tissue formation.

In conclusion, this study demonstrates that 80% of chondrocytes can be replaced by either *h*AMSCs or *h*BMSCs without influencing cartilage matrix production nor stability. Besides, our results support a general trophic role for *h*AMSCs and *h*BMSCs on chondrocytes in co-culture that does not need direct cell-cell contact. These data provide information that can be used to further optimize cell-based cartilage repair.

## Supporting information

S1 Fig Cartilage matrix formation in constructs containing *h*MSCs and/or *b*ACs, 5 weeks after *in-vitro* culture. **(A)** The DNA content of none of the constructs had changed compared to their initial DNA content prior to cell-culture (dotted line). Biochemical evaluation of the GAG **(B)** and collagen **(C)** content, 5 weeks after culture in alginate. The left graphs demonstrate the amount of matrix components per construct, whereas for the right graphs matrix production is normalized to the initially seeded primary ACs. A control condition—containing similar amounts of *b*ACs (0.8*10^6^ nc/ml) without supplementation of *h*MSCs—was evaluated to determine the additional effect of *h*MSCs (3.2*10^6^ nc/ml) on *b*ACs in co-cultures (dotted line). *** indicates a p-value smaller than 0.001 compared to the control condition. Data are shown as mean ± SD. For statistical evaluation, a mixed model was used followed by a Bonferroni's post-hoc comparisons test. *h*AMSC = human Adipose-tissue-derived Mesenchymal Stem Cell (*n = 3* experiments with 3 independent donors); *h*BMSC = human Bone-marrow-derived Mesenchymal Stem Cell (*n = 3* experiments with 3 independent donors); *b*AC = bovine Articular Chondrocyte (*n = 3* experiments with 3 pools of donors). Per experiment, 3 samples were used for analyses.
(TIF)

S2 Fig Macroscopic appearance and histochemical analyses of constructs containing *h*MSCs and/or *b*ACs, 8 weeks after subcutaneous implantation in mice. Macroscopic appearance (top row) of cartilage constructs, as well as a Safranin-O histochemical staining (bottom rows), 8 weeks after subcutaneous implantation. *h*AMSC = human Adipose-tissue-derived Mesenchymal Stem Cell (*n = 3* experiments with 3 independent donors); *h*BMSC = human Bone-marrow-derived Mesenchymal Stem Cell (*n = 3* experiments with 3 independent donors); *b*AC = bovine Articular Chondrocyte (*n = 3* experiments with 3 pools of donors). Per experiment, 2 samples were used for analyses.
(TIF)
