# Supplementary figures and images for: Trophic effects of adipose-tissue-derived and bone-marrow-derived mesenchymal stem cells enhance cartilage generation by chondrocytes in co-culture
Source: PLoS One. 2018 Feb 28;13(2):e0190744. doi: 10.1371/journal.pone.0190744 (PMC5830031; doi:10.1371/journal.pone.0190744)

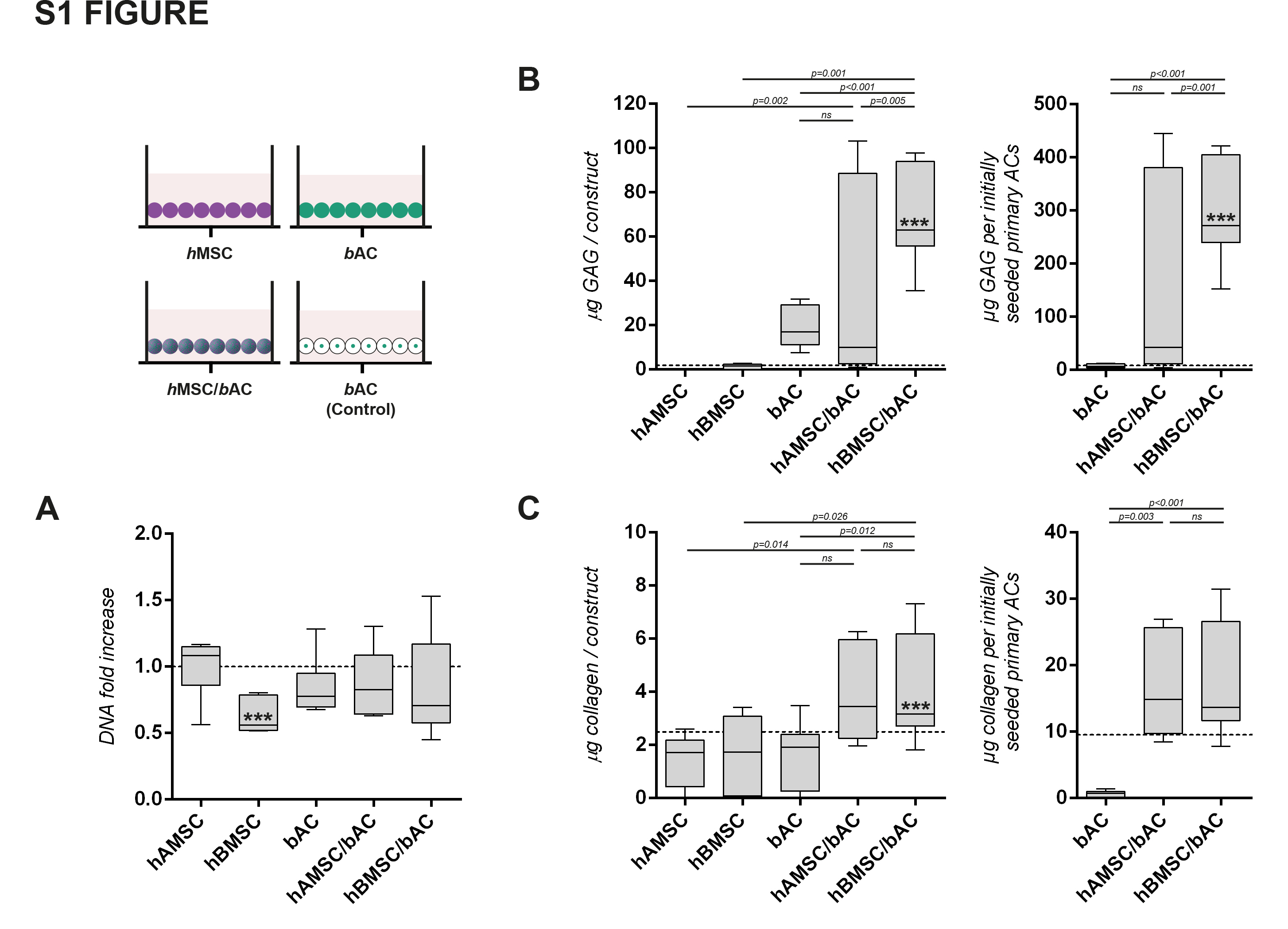

Supplement: S1 Fig — (A) The DNA content of none of the constructs had changed compared to their initial DNA content prior to cell-culture (dotted line). Biochemical evaluation of the GAG (B) and collagen (C) content, 5 weeks after culture in alginate. The left graphs demonstrate the amount of matrix components per construct, whereas for the right graphs matrix production is normalized to the initially seeded primary ACs. A control condition—containing similar amounts of bACs (0.8*106 nc/ml) without supplementation of hMSCs—was evaluated to determine the additional effect of hMSCs (3.2*106 nc/ml) on bACs in co-cultures (dotted line). *** indicates a p-value smaller than 0.001 compared to the control condition. Data are shown as mean ± SD. For statistical evaluation, a mixed model was used followed by a Bonferroni's post-hoc comparisons test. hAMSC = human Adipose-tissue-derived Mesenchymal Stem Cell (n = 3 experiments with 3 independent donors); hBMSC = human Bone-marrow-derived Mesenchymal Stem Cell (n = 3 experiments with 3 independent donors); bAC = bovine Articular Chondrocyte (n = 3 experiments with 3 pools of donors). Per experiment, 3 samples were used for analyses. (TIF) [file pone.0190744.s001.tif]

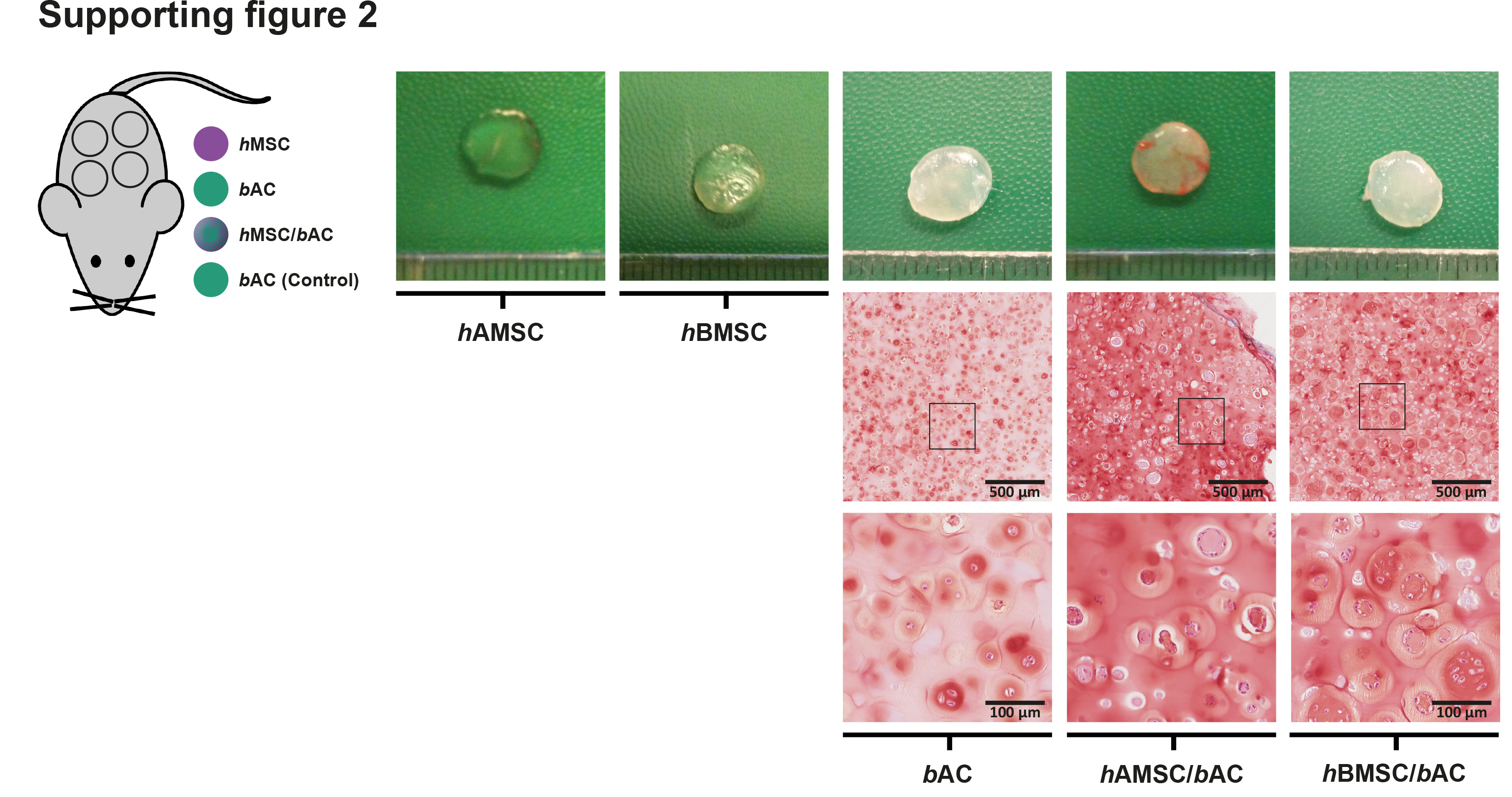

Supplement: S2 Fig — Macroscopic appearance (top row) of cartilage constructs, as well as a Safranin-O histochemical staining (bottom rows), 8 weeks after subcutaneous implantation. hAMSC = human Adipose-tissue-derived Mesenchymal Stem Cell (n = 3 experiments with 3 independent donors); hBMSC = human Bone-marrow-derived Mesenchymal Stem Cell (n = 3 experiments with 3 independent donors); bAC = bovine Articular Chondrocyte (n = 3 experiments with 3 pools of donors). Per experiment, 2 samples were used for analyses. (TIF) [file pone.0190744.s002.tif]
